# Pharmacogenomics of Hypertension Treatment

**DOI:** 10.3390/ijms21134709

**Published:** 2020-07-01

**Authors:** Jacek Rysz, Beata Franczyk, Magdalena Rysz-Górzyńska, Anna Gluba-Brzózka

**Affiliations:** 1 Department of Nephrology, Hypertension and Family Medicine, Medical University of Lodz, 90-549 Lodz, Poland; jacek.rysz@umed.lodz.pl (J.R.); bfranczyk-skora@wp.pl (B.F.); 2 Department of Ophthalmology and Visual Rehabilitation, Medical University of Lodz, 90-549 Lodz, Poland; mrs-89@o2.pl

**Keywords:** pharmacogenetics, polymorphisms, response to drugs, diuretics, ACE inhibitors, angiotensin II receptor blockers, calcium channel blocking (CCB) agents

## Abstract

Hypertension is one of the strongest modifiable cardiovascular risk factors, affecting an increasing number of people worldwide. Apart from poor medication adherence, the low efficacy of some therapies could also be related to inter-individual genetic variability. Genetic studies of families revealed that heritability accounts for 30% to 50% of inter-individual variation in blood pressure (BP). Genetic factors not only affect blood pressure (BP) elevation but also contribute to inter-individual variability in response to antihypertensive treatment. This article reviews the recent pharmacogenomics literature concerning the key classes of antihypertensive drugs currently in use (i.e., diuretics, β-blockers, ACE inhibitors, ARB, and CCB). Due to the numerous studies on this topic and the sometimes-contradictory results within them, the presented data are limited to several selected SNPs that alter drug response. Genetic polymorphisms can influence drug responses through genes engaged in the pathogenesis of hypertension that are able to modify the effects of drugs, modifications in drug–gene mechanistic interactions, polymorphisms within drug-metabolizing enzymes, genes related to drug transporters, and genes participating in complex cascades and metabolic reactions. The results of numerous studies confirm that genotype-based antihypertension therapies are the most effective and may help to avoid the occurrence of major adverse events, as well as decrease the costs of treatment. However, the genetic heritability of drug response phenotypes seems to remain hidden in multigenic and multifactorial complex traits. Therefore, further studies are required to analyze all associations and formulate final genome-based treatment recommendations.

## 1. Introduction

Hypertension is one of the strongest modifiable risk factors for cardiovascular diseases, and prevalence is constantly increasing worldwide [1]. According to estimates, over 116.4 million individuals are suffering from this disease, which is associated with 2303 deaths from cardiovascular disease (CVD) every day [2]. Within the next 20 years, the number of individuals suffering from hypertension is believed to rise by 60% to a total of more than 1.5 billion individuals [1,3]. Currently, 46 percent of U.S. adults are estimated to have hypertension, among whom only 80 percent are aware of their condition, and even fewer follow pharmacological treatment [4,5]. Adequate blood pressure control is reported in approximately 53% of these individuals [4,5]. The high prevalence of undiagnosed and uncontrolled hypertension is associated with a frequent lack of specific clinical manifestations of hypertension (it is also called a “silent killer”), poor efficacy of treatments, and low adherence resulting from possible adverse reactions to active ingredients, despite increasing public awareness of hypertension and its complications [4,5,6]. The low efficacy of some therapies could be related to inter-individual genetic variability. Indeed, genetic studies of families have suggested that heritability accounts for 30% to 50% of inter-individual variation in blood pressure (BP) [7,8,9,10]. Genome-wide studies have confirmed that genetic factors are related not only to blood pressure elevation but also to inter-individual variability in response to antihypertensive treatment [11]. Due to the polygenic nature of hypertension, a single locus cannot be used as a relevant clinical target for all individuals; therefore, the analysis of complex traits, such as drug response phenotypes, should involve the assessment of interactions among multiple loci [12,13]. Genetic variability may modulate the drug response via genes that are engaged in the pathomechanisms of hypertension development and alter the effects of drugs, modifications of mechanistic interactions between drugs and genes, and the polymorphisms in genes related to drug transporters. Moreover, drug responses can be affected by genetic polymorphisms in genes encoding drug-metabolizing enzymes and pleiotropic genes participating in metabolic reactions and complex cascades [4,14]. Genome-wide association studies (GWASs) have led to the discovery of variants associated with drug efficacy and adverse drug reactions. However, due to the multigenic and multifactorial nature of the drug response phenotypes, further research in this area is required to establish reliable recommendations [12,15]. Below, we summarize the most common polymorphisms in genes affecting drug response.

## 2. Calcium Channel Blocking Agents

### 2.1. Mechanism of Calcium Channel Blocker Action 

**Figure 1:**
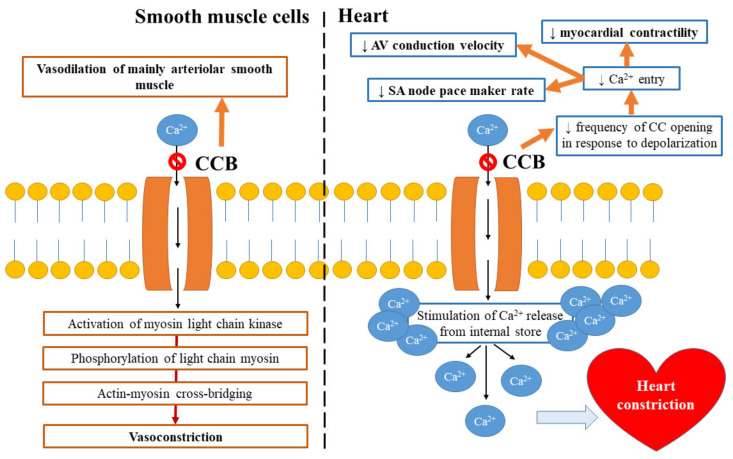
Mechanism of calcium channel blockers’ (CCBs) action.

Calcium channel blockers (CCBs) are drugs that bind to and block mainly the L-type calcium channels present on cardiac and vascular smooth muscle cells (SMCs) [16]. They prevent calcium ions from entering into vascular smooth muscles, which results in muscle relaxation and vasodilation and leads to a decrease in vascular resistance and, consequently, diminished arterial BP [17]. Each subclass of calcium channel blocking agent binds at a specific location. For example, dihydropyridines (e.g., amlodipine and nifedipine) exert vascular selectivity, verapamil has cardiac selectivity, and diltiazem can act in both the heart and blood vessels [1,18]. Figure 1 presents the mechanism of CCB’s action.

### 2.2. Polymorphisms in Genes Affecting CCB Response 

Numerous studies have indicated that the single-nucleotide polymorphisms (SNPs) in genes encoding different ion channels, including the large-conductance voltage and calcium-sensitive potassium channel β1 (*KCNMB1*), the voltage-gated calcium channels α1C (*CACNA1C*), α1D (*CACNA1D*) and β2 (*CACNB2*), and ERG potassium channel (*KCNH2*), can modify the antihypertensive responses to CCB and the risk of adverse cardiovascular outcomes [1,19,20,21,22,23]. Furthermore, variations in the genes coding the family of HECT domain E3 ubiquitin ligases (NEDD4L) and ATP-binding cassette subfamily B member 1 (ABCB1) have been shown to participate in the regulation of blood pressure (BP) in response to anti-hypertensive drugs [11,16]. The result of the INVEST-GENES study demonstrated that the *KCNMB1* genotype can influence responses to verapamil SR in White, Hispanic, and Black hypertensive patients with cardiovascular disease (CAD) [19]. The *KCNMB1* gene encodes the β1 subunit of the large-conductance potassium (BKor Maxi-K type) channel. It has been shown that reduced function of this protein is associated with diminished calcium sensitivity, higher BP, and cardiac hypertrophy [19,24]. Two nonsynonymous polymorphisms in the *KCNMB1* gene—Glu65Lys (rs11739136) and Val110Leu (rs2301149)—were found to modulate inter-patient variability in BP response to verapamil. In Lys65 variant carriers, BP targets were achieved faster (1.47 [interquartile range (IQR 2.77)] months) in comparison to individuals who were homozygous for Glu65 (2.83 [(IQR) 4.17] months; *p* = 0.01). In turn, the Leu110 allele decreased the risk of nonfatal myocardial infarction in patients treated with verapamil but not in those treated with atenolol [19].

Candidate association studies revealed variants in the calcium channels that could facilitate the selection of a CCB or β-blocker—for example, in *CACNA1C*, which encodes the alpha1c-subunit of the L-type calcium channel [24]. Beitelshees et al. [25] screened eight single-nucleotide polymorphisms within *CACNA1C* to search for the relationship between verapamil and atenolol treatment efficacy and the occurrence of primary outcomes of stroke, myocardial infarction, and death. According to the authors, in the presence of the AA genotype, the rate of primary outcomes in individuals treated with verapamil decreased (OR 0.54, 95% CI 0.32–0.92), while the carriers of the GG genotype had a higher risk of a composite primary outcome (OR 4.59, 95% CI 1.67–12.67) [25]. In turn, Bremer et al. [22] studied the efficacy of various CCBs (including amlodipine) in a group of Caucasian individuals with hypertension. They found three variations in *CACNA1C* that significantly affected response to amlodipine and felodipine; two of them, rs22368032 and rs2239050, showed a significant association with uncontrolled hypertension (HTN). However, due to the small sample size and homogeneous population, the author’s results require further confirmation in a larger study of a heterogeneous population. SNPs within the second calcium channel, CACNB2, which encodes the regulatory β2 subunit of the voltage-gated calcium channel, were related to cardiovascular outcomes in subjects randomized to the verapamil arm [26]. White, Black, and Hispanic carriers of the GG genotype of an SNP (rs2357928) within a promoter region had increased risk of the primary outcomes when treated with verapamil compared to those on atenolol. In turn, a Japanese retrospective study of patients with controlled and uncontrolled HTN found three SNPs: rs527974 within *CACNA1C* and rs312481 and rs3774426 within *CACNA1D*, which affected sensitivity to amlodipine and other calcium channel blockers in those with uncontrolled HTN [21,27]. The combined presence of rs312481G/A and rs3774426C/T in *CACNA1D* was associated with a considerable decrease in BP. Subjects with the CC genotype responded better to treatment, presenting lower systolic blood pressure (SBP) [21,27].

Moreover, the SNPs in the enzymes responsible for drug metabolism were found to influence response to drugs. A splicing defect resulting from the presence of polymorphism (14690G>A) within exon 7 of the *CYP3A5* gene (CYP3A5*6, rs10264272), which leads to the deletion of exon 7 and is associated with reduced enzyme activity, has been associated with an altered response to antihypertensive drugs [28]. Langaee et al. [29] used a haplotype approach to investigate the effect of CYP3A5 mutations on BP responses to verapamil in Black, Hispanic, and White individuals. In their study, blood pressure responses to verapamil, to a small extent, depended on the number of *CYP3A5* functional alleles in the first two populations, but not in the third one; carriers of two functional alleles showed the worst response to the treatment [16,29]. The activity of the cytochrome P450 3A4/5 (CYP3A4/5) enzymes is also important for amlodipine, which is a first-line long-acting CCB that inhibits vasoconstriction and improves blood flow by hampering calcium influx via the L-type calcium channels into vascular SMCs [16,30]. According to past studies, polymorphisms within genes that encode the enzymes participating in amlodipine metabolic break-down into inactive pyridine metabolites (M9) affect this drug’s activity and thus modify patients’ anti-hypertension responses to treatment [16,31]. In one of these studies, a polymorphism in the promoter region of CYP3A4*1B (-392A/G, rs2740574) had prognostic potential to predict blood pressure responses in a population of African American women with early hypertensive nephrosclerosis [32]. Female carriers of the A allele were over three times more likely to reach a target mean arterial pressure (MAP) of 107 mm Hg (*p* = 0.02). It has been suggested that levels of P-glycoprotein (PgP), which is a multidrug transporter mediating the cellular transport of several drugs, may be responsible for the gender-specific response to amlodipine. PgP levels were found to be two-to-three times higher in men than in women. Therefore, the P-glycoprotein-mediated efflux of amlodipine might result in higher intracellular concentrations in women [32]. The enhanced hepatic clearance of CYP3A and P-glycoprotein in women compared to men has been confirmed in multiple studies and might be related to the lower weight and organ size, greater amount of body fat, and lower glomerular filtration rate of women [33]. The presence of intronic polymorphisms within CYP3A4 (T16090C, rs2246709) was considerably associated with a BP response to amlodipine in individuals randomized to a lower MAP group (≤92 mm Hg). Irrespective of gender, the likelihood of reaching the target MAP of 107 mm Hg was two-times higher in carriers of the 16090C allele than in T allele carriers (*p* = 0.01). Due to the fact that a similar effect was not observed in individuals randomized to ramipril, it seems that the relation between the aforementioned CYP3A4 genotype and BP response may be limited to amlodipine [32].

In turn, Kim et al. [34] studied the impact of the CYP3A5*3 (A6986G, rs776746) genotype on both the pharmacodynamics and pharmacokinetics of amlodipine in a group of healthy Korean males. CYP3A5*1 is a functional allele, while the mutation in intron 3 of the CYP3A5*3 variant results in a splicing defect and the formation of non-functional proteins [28]. Kim et al. [34] found a statistically significant difference (20%) in the oral clearance of amlodipine between CYP3A5*1 carriers (27.0 ± 8.2 L/h) and CYP3A5*3/*3 carriers (32.4 ± 10.2 L/h) (*p* = 0.063). Moreover, CYP3A5*1 carriers (3.8 ± 1.1 ng/mL) had a considerably higher peak plasma concentration than CYP3A5*3/*3 carriers (3.1 ± 0.8 ng/mL) (*p* = 0.037); however, there were no significant differences in BP and pulse rate between the studied groups. The authors stated that polymorphisms within the *CYP3A5* gene could modulate the inter-individual disposition of amlodipine [34]. However, according to Zhu et al. [31], CYP3A4 is mainly responsible for amlodipine dehydrogenation, and, therefore, the polymorphisms in the *CYP3A5* gene likely do not exert any effect on the pharmacokinetic variability of amlodipine.

Amlodipine’s bioavailability and concentration can also be influenced by polymorphisms in the transporter, ATP-binding cassette subfamily B member 1 (ABCB1) (or multi drug resistance 1; MDR1) [16]. The *ABCB1* gene encodes the aforementioned glycoprotein P (P-gp)—the drug efflux pump involved in the cellular accumulation of amlodipine [35]. Polymorphisms in exon 26 (C3435T, rs1045642) of the *ABCB1* gene were found to decrease glycoprotein *p* expression and limit its function, thereby altering the absorption and tissue concentrations of a given substrate (e.g., amlodipine) [36]. Zuo et al. [35] demonstrated that the polymorphism C3435T in the *ABCB1* gene might modify the plasma concentrations of amlodipine in hypertensive Han Chinese patients. However, it had no impact on the efficacy of amlodipine treatment. In *ABCB1* 3435CC and 3435CT individuals, the expression of P-gp was greater than that in 3435TT subjects. Moreover, amlodipine’s oral clearance (CL/F) was 1.5-fold higher in carriers of the TT genotype compared to other groups of carriers [35]. Table 1 presents a summary of the effects of polymorphisms on the efficacy/concentration of CCBs.

## 3. Angiotensin-II Receptor Blockers (ARB) and Angiotensin-Converting Enzyme Inhibitors (ACEi)

**Figure 2:**
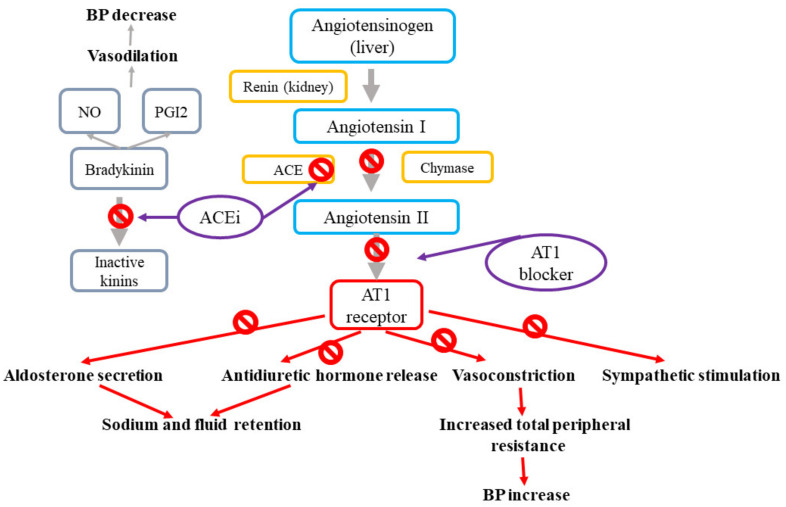
Mechanisms of action of angiotensin-II receptor blockers and angiotensin-converting enzyme inhibitors.

Renin-angiotensin-aldosterone (RAA) system and angiotensin II receptor blockers (ARB) and angiotensin converting enzyme inhibitors (ACEi) action. The renin–angiotensin system modulates blood pressure and sodium homeostasis through effects that are coordinated via combined mechanisms in the kidney, cardiovascular system, and central nervous system [1,37]. The effects of the renin–angiotensin system are mediated by the renin-related conversion of angiotensinogen into angiotensin I, which is further cleaved by the angiotensin-converting enzyme (ACE) to produce angiotensin II (Ang II). Ang II, which is the final effector of the system, stimulates the angiotensin II type 1 receptors (AT1R) present in the vasculature, kidney, and central nervous system, resulting in vasoconstriction, sodium reabsorption, and increased sympathetic tone [37]. The most widely prescribed drugs targeting the renin–angiotensin system involve ACE inhibitors, which impede the formation of angiotensin II, and angiotensin II receptor blockers (ARB), which antagonize the effect of angiotensin II by binding to AT1R [1]. Figure 2 presents the mechanisms of action for angiotensin-II receptor blockers and angiotensin-converting enzyme inhibitors.

### Polymorphisms in Genes Affecting the ARB and ACEi Response 

Numerous studies have suggested that polymorphisms in genes encoding components of the renin–angiotensin-aldosterone system may influence the pharmacogenomics of angiotensin-II receptor blockers and angiotensin-converting enzyme inhibitors. The beneficial effects of *ARB* are associated with several pleiotropic effects, including effects resulting from vasodilation related to the nitric oxide (NO) produced by endothelial NO synthase (NOS3) [38,39]. Various studies have indicated the pharmacogenomics relevance of NOS3 for ACEi and ARB responses [1]. Mason et al. [40] demonstrated that endothelial cells homozygous for the C allele (the −786T/C polymorphism in NOS3, rs2070744) can respond to ARB olmesartan treatment with more enhanced NO formation than heterozygous cells. The authors concluded that hypertensive subjects carrying the C allele might have better responses to enalapril and olmesartan. Moreover, Oliveira-Paula et al. [41] observed that the T allele for the NOS3 −665C/T SNP (rs3918226) is associated with greater responses to enalapril. An opposite effect was demonstrated for the A allele of the NOS3 tagSNP (rs3918188) and the CAG haplotype involving NOS3 tagSNPs [41]. A meta-analysis using data on the losartan response from GENRES and SOPHIA studies and data concerning candesartan from a GERA II study revealed that rs4953035, which is located near the LRPPRC region that codes for the mitochondrial leucine-rich PPR (pentatricopeptide repeat) motif-containing protein (expressed in many tissues, including the heart and kidneys), has an association with systolic BP responses [7]. In turn, a GWAS analysis found that hypertensive carriers of the GG genotype for a polymorphism (rs10752271) located within a gene encoding calcium/calmodulin dependent protein kinase 1D (CAMK1D) (which participates in aldosterone synthesis) have better BP responses to losartan [42]. Other GWAS analyses comprising individuals from the GERA study identified three loci influencing the antihypertensive response to candesartan: The SNP rs11020821 near the *FUT4* gene (encoding fucosyltransferase 4), rs11649420 in the *SCNN1*G gene (encoding sodium channel, non-voltage-gated 1, gamma subunit), and rs3758785 in the *GPR83* gene (encoding G protein-coupled receptor 83) [43]. This study revealed that the GG genotype for the rs11649420 polymorphism in the gene encoding the γ-subunit (SCNN1G) of the amiloride-sensitive sodium channel is associated with a three-fold greater BP response to candesartan compared to a combined group of AA+AG. SNPs within SCNN1G were found to be the most strongly related to BP. However, their relationship with the BP response to hydrochlorothiazide showed an opposite effect; such an effect could be explained by the counter-regulatory mechanism involving sodium reabsorption as a way to maintain intra-arterial volume and prevent decreases in BP after inhibition of the renin–angiotensin system with candesartan [43]. Moreover, the authors showed that in rs3758785 GG carriers, the odds of having a good BP response to candesartan are over 16-fold greater compared to AA carriers. However, the aforementioned significant association was markedly diminished following adjustment for rs11020821 near FUT4. Further, the SNPs in the gene responsible for aldosterone synthesis could affect the drug response. The SNP −344C/T (rs179998) in the *CYP11B2* gene encoding aldosterone synthase, which catalyzes the final step of aldosterone production in juxtaglomerular cells, was shown to modulate aldosterone level, hypertension susceptibility, and blood pressure responses to ARB [44,45,46,47]. However, the results for this polymorphism are conflicting, since in some studies, the C allele of this SNP was related to blood pressure response, while in others, the BP lowering effect was attributed to the T allele [47,48,49]. In turn, a randomized, double-blind, crossover, placebo-controlled GENRES study identified a missense variant (349G/A, rs3814995) within the coding region of the *NPHS1* gene that is significantly associated with responses to losartan. This SNP produces an amino acid substitution of glutamic acid to lysine (p.Glu117Lys) in the nephrin, which is a transmembrane protein and structural component of the slit diaphragm in the kidney, as well as an important contributor to blood pressure regulation [1,7]. The presence of rs3814995 was associated with systolic (*p* = 0.03) and diastolic (*p* = 0.02) BP responses in GERA II and diastolic BP responses (*p* = 0.03) in SOPHIA [7].

The mechanism of angiotensin-converting enzyme inhibitors (ACEis) involves the reduction of angiotensin II formation and indirect vasodilation produced by nitric oxide (NO). Due to the fact that PKCα signaling seems to be involved in both mechanisms, its relationship with drug response was previously studied [12]. Polymorphism rs16960228 in the protein kinase C alpha (PRKCA) gene is associated with BP responses in hypertensive patients classified as poor or good responders to enalapril. Oliveira-Paula et al. [50] found that GACAA genotypes and the A allele are associated with a lower reduction in mean BP and DBP after treatment and, therefore, with worse responses to enalapril compared to the GG genotype (*p* < 0.05). In turn, Turner et al. [51] observed that hypertensive carriers of the GG genotype have significantly lower PRKCA expression compared to individuals with GACAA genotypes. It could be hypothesized that the presence of the GG genotype for the rs16960228 polymorphism can stimulate responses to drugs enhancing PKCα, including ACEi [50]. However, it is also possible that PKCα signaling is less influenced by ACEi in carriers of the GA+AA genotypes and, therefore, that their response to such drugs is worse or that responses to enalapril might be influenced by interactions among genes within the ACEi pathway [12,50]. In another study, Silva et al. [52] demonstrated that TC/CC genotypes and the C allele for the endothelial nitric oxide synthase (eNOS, NOS-3) -786T/C (rs2070744) polymorphism are more frequent in good responders to enalapril treatment than in those who responded worse. Also, the TT genotype for the bradykinin receptor B2 (BDKRB2) -58C/T (rs1799722) polymorphism occurred more frequently in individuals who responded better to enalapril compared to poor responders. Studies on the combined presence of polymorphisms in BDKRB2 and NOS3 have demonstrated their involvement in antihypertensive responses to enalapril [41,52]. A gene–gene interaction analysis revealed that the combined presence of the CC genotype for the rs1799722 SNP in the BDKRB2 and TC genotype for the rs2070744 SNP in the promoter region of the *NOS3* gene is associated with a better response to enalapril treatment [52]. In a combined analysis of PRKCA, BDKRB2, and NOS3 performed by Oliveira-Paula et al. [50], the above-mentioned relationship was statistically significant in the presence of the GG genotype for the rs16960228 SNP in the *PRKCA* gene. This finding may be associated with the fact that PKCα enhances the transcription of the *NOS3* gene, upregulates eNOS activity, and thus promotes increased NO production and vasodilation. ACEi also stimulates the bradykinin receptors on endothelial cells, thereby increasing tissue bradykinin levels, which may lead to PKCα-mediated NOS3 upregulation [50]. Therefore, apart from the presence of polymorphisms in eNOS or in genes that contribute to NOS3 activation gene–gene interactions could also modulate the responses to ACE inhibitors and ARB. The summary of the results of the most interesting studies on angiotensin-II receptor blockers and angiotensin-converting enzyme inhibitors are presented in Table 2.

## 4. β-Adrenergic Antagonists (β-Blockers)

### 4.1. Effects of β-Adrenergic Blocker Actions 

**Figure 3:**
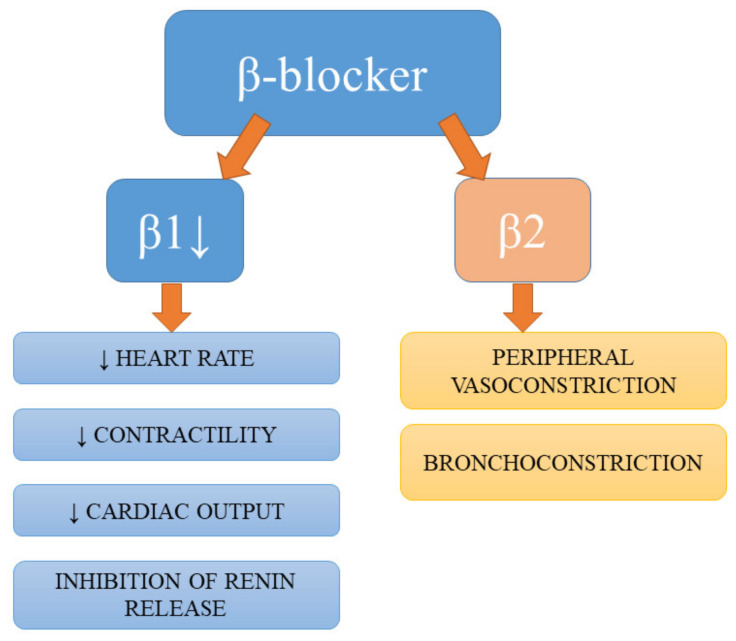
Effects of action of β-blockers.

Despite not being a first-line antihypertensive pharmacotherapy, according to the Joint National Committee (JNC8) hypertension guidelines [53], β-blockers are still widely prescribed and used in certain subgroups of patients [1]. Apart from the antihypertensive effects of β-blockers resulting from the blockage of their targets on the juxtaglomerular cells of the kidney, the reduction in renin secretion, and subsequent hampering of circulating angiotensin II production, these drugs also diminish myocardial contractility, heart rate, and cardiac output [18,54]. Studies have demonstrated the beneficial effects of β-blockers on endothelial dysfunction and indicated their involvement in the endothelial and vasculature-related mechanisms of BP-lowering [55,56]. The summary of effects of action of β-blockers are presented in Figure 3.

### 4.2. Polymorphisms in Genes Affecting the Adrenergic Receptor Blocker Response 

The major protein target of all β-blockers is the β1-adrenergic receptor encoded by the *ADRB1* gene [1,57]. Numerous studies have searched for polymorphisms within the *ADRB1* gene that could affect the function of its protein product or modulate its response to drugs. Two polymorphisms (rs1801252: Ser49Gly and rs1801253: Arg389Gly) have been demonstrated to influence intracellular signaling mediated by the β1-adrenergic receptor [58]. Both the Ser49 and Arg389 alleles boosted intracellular responses to β1-adrenergic receptor agonists compared to the other variant alleles [58]. The observation of the differential responses to β-blockers among Black and Caucasian populations laid the basis for GWAS analyses targeted at finding ethnicity-associated polymorphisms. The INVEST-GENES study, which comprised an ethnically diverse, elderly population of patients with hypertension and documented coronary artery disease (CAD), demonstrated that the ADRB1 Ser49-Arg389 haplotype, which affects agonist-mediated downregulation and signaling activity, is associated with a considerable risk of all-cause death (odds ratio 3.66, 95% CI 1.68–7.99), irrespective of the number of alleles present (1 or 2) [59]. Mortality risk was more evident in individuals treated with verapamil. However, this association lost its significance in subjects receiving atenolol. According to the authors, individuals with the Ser49-Arg389 haplotype should preferentially receive β-blocker therapy because it decreased the risk of mortality. In contrast, the results of the Secondary Prevention of Small Subcortical Strokes (SPS3) trial indicated that in atenolol treated carriers of the Gly49 allele (ADRB1), the risk of major adverse cardiovascular events was higher (hazard ratio, HR 2.03; 95% CI 1.20–3.45); therefore, the authors suggested that these patients should preferentially receive CCB therapy [60]. In turn, Johnson et al. [61] showed that BP responses to metoprolol among White, African American, and Hispanic carriers of the Arg/Arg genotype (Arg389Gly) were better compared to those of the Gly allele carriers. Further, in Chinese hypertensive 389Arg/Arg patients, treatment with a β-blocker (carvedilol) reduced blood pressure to a greater extent than in individuals carrying the Gly allele [62]. However, Chen et al. [63] observed that subjects carrying the Gly/Gly genotype for the Arg389Gly polymorphism showed greater antihypertensive responses to metoprolol, while two European prospective studies failed to find any association between the Arg389Gly polymorphism and blood pressure responses to β-blockers [64,65]. Adrenergic signal transduction mediated through adrenergic receptors and further via the G protein pathway is of key importance for rapid adjustment to increased cardiovascular demands. Therefore, the effects of polymorphisms within G protein–coupled receptors (GPCRs) on responses to drugs have been widely studied. The PEAR and INVEST cohorts identified polymorphisms within the *GRK4* gene, which modulated atenolol mediated BP reduction and cardiovascular outcomes [66]. *GRK4* plays a role in BP homeostasis via the phosphorylation of GPCRs, which are vital for BP regulation, and potentially via β1-adrenergic receptors—the key protein targets of β-blockers [67]. The role of the following nonsynonymous SNPs in *GRK4*, A142V (rs1024323) and R65L (rs2960306), within the GPCR interacting region of *GRK4*, as well as the A486V (rs1801058) SNP resulting in G to T substitution within the membrane targeting region, have also been studied [67]. All aforementioned SNPs likely represent a gain in functional polymorphisms that improve the ability of GRK4 to bind to, phosphorylate, and desensitize GPCRs. Vandell et al. [66] demonstrated that GRK4 65L and 142V variants, as well as the presence of the 65L-142V haplotype significantly reduce responses to β-blocker monotherapy and also enhance the risk of adverse long-term CV outcomes. GRK4 65L and 142V are associated with reduced BP responses to atenolol in both Caucasians and African Americans [66]. In a genome-wide association study of metoprolol treated individuals from the Pharmacogenomic Evaluation of Antihypertensive Responses-2 (PEAR-2) study and atenolol treated subjects from the PEAR study, rs294610 (an intronic SNP resulting in C/A transversion) in the *FGD5* gene (encoding FYVE, RhoGEF, and the PH domain containing protein 5) was found to significantly improve BP response following metoprolol and atenolol treatment (the latter analysis was carried out in an independent cohort of European Americans) [68]. Carriers of the A allele had considerably better BP responses than non-carriers. Other GWAS studies have identified the BP-related phenotypes of FGD5. A large study enrolling European American hypertensive patients found that the loci within FGD5 are associated with DBP and SBP. The Welcome Trust Case Control Consortium, using an intermediate approach between the genome-wide and candidate gene methods to analyze the link between hypertension and genes expressed in the endothelium, identified FGD5 as one of the possible loci [69]. According to the authors, this gene may be involved in the development and progression of hypertension through its role in vascular remodeling. Animal studies confirmed the regulatory role of FGD5 in endothelial cell-specific apoptosis and its effect on vascular pruning [70]. Moreover, in vitro studies using human cell lines have provided evidence for the involvement of FGD5 in the proangiogenic action of the endothelial growth factor, demonstrating the potential role of FGD5 in the development/progression of vasculature-related diseases, including hypertension [71]. However, further studies are necessary to understand the functional role of SNPs within the *FGD5* gene in improved BP responses to β-blockers. In turn, in an analysis of the results obtained from two studies (PEAR-2 and PEAR) with an independent atenolol add-on therapy, Singh et al. [68] identified rs45545233 polymorphism in the *SLC4A1* gene, which was significantly associated with a reduced BP response to β-blockers. The PEAR-2 metoprolol-treated cohort analysis also found the *SLC4A1* gene encoding for glycoprotein in the plasma membrane (the band 3 anion transporter belonging to the anion exchanger family) to be related to drug response. SLC4A1 is mainly expressed in the erythrocyte membrane and the collecting ducts of the kidney, where it is involved in the electro–neutral exchange of HCO_3_^−^ for Cl^−^ and the transport of glucose and water [68]. According to Kokubo et al. [72], polymorphisms in SLC4A1 are significantly associated with hypertension and BP variation in the Japanese population. However, the exact mechanism of this relationship needs to be better understood. Gong et al. [73] observed that a deletion in the intronic region of the *SLC25A31* gene (rs201279313) in hypertensive African Americans treated with β-blockers is associated with better antihypertensive responses to β-blockers when compared to subjects carrying two wild-type alleles. The *SLC25A31* (Solute Carrier Family 25 Member 31) gene encodes ADP/ATP translocase 4, which catalyzes ADP/ATP exchange across the mitochondrial membranes and regulates membrane potential. Gong et al. [73] also demonstrated a better BP response to β-blockers in subjects carrying the deletion allele of rs11313667 within the intronic region of the *LRRC15* gene (encoding leucine rich repeat containing 15) compared to carriers of two wild-type alleles [74]. The prospective, randomized Nordic Diltiazem (NORDIL) study, which enrolled over 10,000 subjects aged 50–74 from Norway and Sweden, found that the intronic SNP (rs12946454) of *PLCD3* is involved in modulating the response to diltiazem [75]. The T allele of the *PLCD3* gene, which encodes the phospholipase C enzyme that is vital for calcium release in smooth muscle and maintaining vascular tone, was shown to be associated with increased systolic and diastolic BP responses to diltiazem [76]. Another analysis on the association between four SNPs and atenolol responses in 467 Finnish LIFE (Losartan Intervention For Endpoint reduction in hypertension study) patients revealed that only rs2514036, a variation at the transcription start site of the *ACY3* gene (coding for aminoacylase III), altered BP’s response to atenolol in men [74]. In turn, three SNPs, rs2514036, rs948445, and rs2514037, in the *ACY3* gene were found to exert an impact on BP responses to bisoprolol in Caucasians [7].

Polymorphisms in genes not only modulate the pharmacodynamics of β-blockers but can also regulate their pharmacokinetics [1]. CYP2D6, a key cytochrome enzyme involved in the metabolism of β-blockers, seems to be a candidate gene [77]. Based on sound evidence indicating that CYP2D6 genotypes alter BP responses to β-blockers, the Pharmacogenetics Working Group of the Royal Dutch Pharmacists Association has established recommendations on the therapeutic dose of metoprolol depending on the CYP2D6 genotype [78]. However, the usefulness of CYP2D6 genotyping as a guide for metoprolol therapy in hypertensive individuals has been challenged by the results of some other studies [79,80,81]. A summary of the results of most interesting studies on the β-adrenergic antagonists is presented in Table 3.

## 5. Diuretics

**Figure 4:**
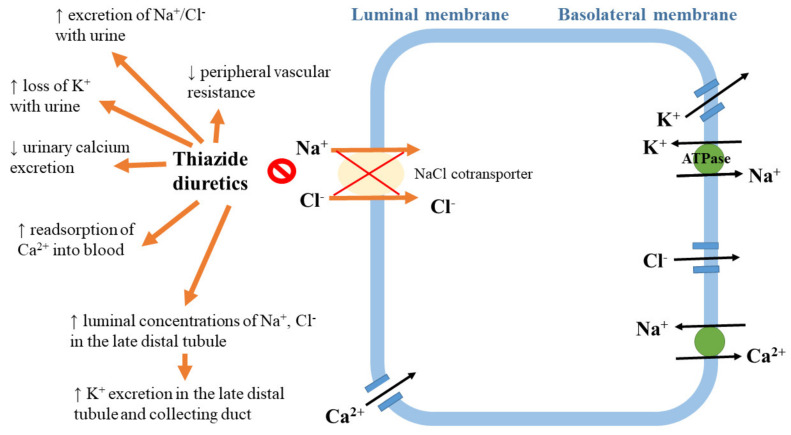
Effects of action of thiazide diuretics.

*The mechanism of diuretic action.* Diuretics, especially thiazide and thiazide-like diuretics, are the first-line drugs of choice for the majority of patients with hypertension [1,53]. The thiazide diuretic hydrochlorothiazide inhibits the sodium chloride cotransporter expressed in the distal convoluted tubule of the nephron [82]. The initial antihypertensive effects of these drugs involve the enhancement of sodium excretion (natriuresis) and the diminishing of extracellular volume, which results in a reduction in cardiac output. Moreover, these drugs exert long-term effects via a decrease in vascular resistance, possibly resulting from an inhibition of the sympathetic nervous and/or renin–angiotensin systems [83]. Effects of action of thiazide diuretics are summarized in Figure 4.

### Polymorphisms in Genes Affecting Diuretic Response 

As diuretics’ effects are realized via different mechanisms, several candidate genes have been suggested to influence individual responses to these drugs [1]. The effectiveness of the hydrochlorothiazides (HCTZs) used in monotherapy can be reduced by factors associated with inter-individual variation, which can lead to increased mortality among patients with uncontrolled hypertension [16,84,85]. Moreover, thiazides may cause hypokalemia, impair glucose tolerance, or increase serum cholesterol and uric acid levels; susceptibility to the abovementioned adverse reactions might also be related to inter-individual variation, age, gender, and ethnicity [86]. It has been demonstrated that SNPs within 3-hydroxy-3-methylglutaryl-CoA synthase (HMGCS) in African Americans and Caucasians are associated with elevated blood glucose levels after treatment with both chlorthalidone and HCTZ [87,88]. A meta-analysis of four studies and over 1000 patients revealed that the polymorphisms within the *ACE* and *ADD1* genes affected blood pressure responses to HCTZ [89]. The *ADD1* gene encodes α-adducin, which is a cytoskeleton-related protein modulating ion transport, while the *ACE* gene encodes the angiotensin-converting enzyme, a central component of the renin–angiotensin system that regulates the volume of body fluids and controls blood pressure [1,90,91]. In a meta-analysis performed by Choi et al. [85], a significant association was observed between the ACE II and DD genotypes and blood pressure changes (standard differences in means = 0.256; 95% CI, 0.109–0.403). Moreover, Sciarrone et al. [92] demonstrated that carriers of the II genotype showed better antihypertensive responses to hydrochlorothiazide than those carrying the DD genotype. A study on the Han Chinese population revealed that this polymorphism modulated hydrochlorothiazide responses in a gender-specific manner. In men carrying DD genotype, the effects of antihypertensive therapy were better than those in women carrying the II genotype [56]. However, other studies failed to show such an association [93,94]. In a GenHAT (Genetics of Hypertension Associated Treatments) study, which was an ancillary to Antihypertensive and Lipid-Lowering Treatments to Prevent Heart Attack Trial (ALLHAT), several candidate hypertension-related genes were analyzed in 39,114 individuals in order to determine the possible variants of six genes affecting antihypertensive drug response [94]. The results of GenHAT indicated that the DD genotype (*ACE*; rs1799752) does not influence BP reduction or cardiovascular outcomes in patients on ACE-inhibitor therapy compared to ID and II alleles. For the *ADD1* Gly460Trp polymorphism (rs4961), a considerable relationship was revealed for the genotypes of GlyGly vs. GlyTrp (standard differences in means = 2.78; 95% CI, 0.563–4.99) and GlyGly vs. TrpTrp (standard differences in means = 1.80; 95% CI, 1.38–2.22) [89]. Another study indicated that carriers of the Trp allele for the Gly460Trp polymorphism in the *ADD1* gene have diminished baseline plasma renin activity and better antihypertensive responses to hydrochlorothiazide treatment compared to Gly/Gly homozygotes [26]. Glorioso et al. [95] suggested that this polymorphism (rs4961) might modulate renal sodium handling by changing ion transport across the cell membrane. Some other studies indicated that *GNB3***,** which encodes the β3-subunit of the G-protein, is another gene possibly involved in responses to hydrochlorothiazide treatment [96]. This family of proteins participates in signal transduction from the membrane receptors to a wide range of intracellular effectors [1,96]. The presence of the T allele for the C825T (rs5443) polymorphism in the *GNB3* gene is associated with the formation of an RNA splice variant lacking nucleotides 498–620 within exon 9, which results in structural modifications of the β3-subunit of the G-protein and modulation of signal transduction [97]. Turner et al. [98] found that the T allele is associated with better antihypertensive responses to hydrochlorothiazide and that this effect is gene–dose related. Since a larger study provided conflicting results [55], the association between the rs5443 polymorphism and hydrochlorothiazide responses requires confirmation.

In a study analyzing the effects of SNPs on the responsiveness of 228 male patients from European decent to four classes of anti-HTN drugs, including HCTZ, over 80 different polymorphisms were identified [7]. However, a significant association was found only for aldehyde dehydrogenase 1 family member 13 (ALDH1A3) and chloride intracellular channel 5 (CLIC5). The authors suggested that two other family members of the ADH gene (*ALDH1A2* and *ALDH7*) are related to the presence of hypertension in African Americans, while ALDH2 was associated with BP control in an East Asian population [7,99,100]. A GWAS analysis found a relationship between the SNP rs261316 in the *ALDH1A2* gene and uncontrolled blood pressure following treatment with a thiazide diuretic/β-blocker combination in white participants of the PEAR study [101]. The results of the INVEST study confirmed the aforementioned findings [101]. Other GWAS studies identified various additional loci associated with BP responses to HCTZ in different ethnic groups [16,102,103]. In African American populations, SNPs within the lysozyme (LYZ), Yeast Domain Containing 4 (YEATS4), and fibroblast growth receptor substrate 2 (FRS2) genes located on chromosome 12q15 were shown to exert an effect on the HCTZ response [103]. African Americans carrying the ATC haplotype (a combination of alleles for SNPs rs317689 (A), rs315135 (T), and rs7297610(C)) were demonstrated to respond to HCTZ much better than in persons with ACT or ATT haplotypes [103]. However, in the PEAR study [104] the ATT haplotype in the African American population was also associated with a good HCTZ response. Moreover, a reduction in YEATS expression was observed in African Americans who were CC homozygotes for the SNP rs7297610, but not in T carriers, which implies an association between the YEATS variant and the HCTZ response [16,102,104]. The results of a large meta-analysis (six clinical trials within the International Consortium for Antihypertensive Pharmacogenomics Studies) concerning the BP response to hydrochlorothiazide in White hypertensive individuals reported a strong correlation between hydroxy-delta-5-steroid dehydrogenase, 3 β- and steroid δ-isomerase 1 gene (*HSD3B*1), and BP response (*p* < 2.28×10^-4^). HSD3B1 encodes the 3β-hydroxysteroid dehydrogenase enzyme, which is of key importance in the biosynthesis of aldosterone and endogenous ouabain [105]. Other studies also demonstrated the relationship between genetic variants in HSD3B1 and HTN or BP variation [106,107,108]. In these studies, the CC genotype at rs6203 was related either to the presence of hypertension [109] or to higher blood pressure values [106,107,108]. In turn, Svensson-Farbon et al. [110] demonstrated that a genetic polymorphism (rs4149601G/A) in neural precursor cell expressed, developmentally down-regulated 4-like, E3 ubiquitin protein ligase (NEDD4L), which led to the formation of a cryptic splice site in NEDD4L. Numerous other studies have shown that polymorphisms in the *NEDD4L* gene influence salt sensitivity, plasma renin concentrations, and susceptibility to develop hypertension [111,112,113]. The results of the NORDIL (Nordic Diltiazem) Study, which enrolled Caucasian hypertensive patients, found that the presence of the G allele downregulated the epithelial sodium channel (ENaC) and further increased sodium retention/reabsorption in the distal nephron, along with the development of hypertension [110]. Moreover, the authors found a relationship between the G allele and a greater BP lowering response to HCTZ in comparison to AA homozygotes [110]. The association between the rs4149601G/A polymorphism and a greater response to thiazides was confirmed in the Pharmacogenomic Evaluation of Antihypertensive Responses (PEAR) study [104]. McDonough et al. [114] also found that white hypertensive carriers of cumulative copies of the G-C haplotype of the *NEDD4L* gene (for SNPs rs4149601 and rs292449, respectively) responded better to hydrochlorothiazide. These observations were not replicated in African Americans [114]. Therefore, further research is needed to determine whether treatment decisions in patients with HTN could be based on analysis of this polymorphism. In turn, the meta-analysis of data from the PEAR-1, GERA-1, NORDIL, and GENRES studies also revealed a genome-wide significance for rs16960228 (A/G) in the protein kinase C alpha gene (*PRKCA*). Turner et al. [51] also demonstrated that in cohorts of European Americans, systolic and diastolic BP responses to HCTZ treatment were consistently greater in carriers of the GACAA genotypes than in homozygote GG carriers, likely due to the fact that the A-allele (rs16960228) is related to higher baseline PRKCA expression.

In the Caucasian population, the BP response to HCTZ monotherapy has also been found to be related to SNPs within the SH2B Adaptor Protein 3 gene (SH2B3—rs3184504), fibroblast growth factor 5 (FGF5—rs1458038), and Early B Cell Factor (EBF1—rs45551053) [16,115]. The first aforementioned SNP was shown to be related to higher BP values and increased risk for HTN in Caucasian individuals [115]. Moreover, carriers of the CC genotype responded better to anti-hypertension drugs (especially atenolol) than other genotypes (TT and TC). The GWAS of variants affecting antihypertensive responses to hydrochlorothiazide further revealed that rs2273359 within the EDN3 region significantly modulates the SBP response to HCTZ. In this study, greater improvements in BP in response to HCTZ treatment were seen in carriers of the CG genotype compared to carriers of the CC genotype [51]. The PEAR and PEAR-2 studies [116] suggested that the antihypertensive response to thiazide diuretics can be associated with genetic variants of protein phosphatase 1 regulatory subunit 15A (PPP1R15A), dual specificity phosphatase 1 (DUSP1), and FBJ murine osteosarcoma viral oncogene homolog (FOS) (*p* <  2.0 × 10^-6^). In those who better responded to HCTZ or chlorthalidone, an up-regulation in the transcription of the aforementioned genes was observed. In turn, a GWAS analysis of two Italian cohorts (Milan Hypertension Pharmacogenomics of hydrochlorothiazide; MIHYPHCTZ and Pharmacogenomics of Hydrochlorothiazide Sardinian Study; PHSS), which assessed the response to HCTZ treatment in Caucasian individuals with an office SBP > 140 mmHg and a DBP > 90 mmHg without prior treatment [116], revealed six variants that are predictive of the SBP response and five variants predictive of DBP [24]. The strongest effect on the SBP response was observed for the intronic polymorphisms within TET2 (Tet methylcytosine dioxygenase 2) (rs12505746) and two SNPs in CSMD1 (CUB and Sushi multiple domains protein 1) (rs7387065 and rs11993031) [116]. CSMD1 belongs to the family of the vacuolar protein sorting-associated protein 13C, while TET2 is involved in *αENaC* gene transcription in the renal collecting duct. Genome-wide prioritization and transcriptomics analyses revealed that SNP rs10995 in the *VASP* gene (encoding the vasodilator-stimulated phosphoprotein) is a functional SNP associated with hydrochlorothiazide responses [77]. It was demonstrated that the G allele for this SNP is related to greater blood pressure responses to hydrochlorothiazide and enhanced mRNA expression of VASP. In turn, the re-sequencing of chromosome 12q in participants from the GERA and PEAR studies lead to the identification of a novel missense SNP (rs61747221) in the *BEST3* gene, which was related to blood pressure responses to hydrochlorothiazide treatment [117]. Better antihypertensive responses to hydrochlorothiazide were reported in individuals carrying the AA+AG genotypes for this polymorphism in comparison to GG carriers [117].

The ethnic differences in response to ACE inhibitors and beta blockers suggest that the development of hypertension may be related to different pathways in various ethnic groups. Clinical observations indicate that, in general, Black populations respond more favorably to diuretics and calcium channel blockers, while White populations respond in a similar manner to all drug classes [118,119]. A summary of the results of the most interesting studies on diuretics is presented in Table 4.

## 6. Conclusions

This article reviewed the recent literature on the chief types of antihypertensive drugs, including β-blockers, ACE inhibitors, ARB, diuretics, and CCB. Due to the numerous studies on this topic and their sometimes contradictory results, the presented data are limited to just several SNPs that alter drug response. The inconsistencies in the studies’ results may be related to interethnic differences in the distributions of the analyzed polymorphisms, the presence of heterogeneous phenotypes, or the type of strategy applied [1].

In the pharmacogenomic studies, two main approaches were used: candidate gene and GWAS analyses. The polygenic nature of hypertension makes the search for the most appropriate SNPs associated with this disorder, as well as the relationships between individual genes and the drug responses in different ethnic groups, very difficult [16]. The above-mentioned approaches may overlook some associations that can only be identified by using combinations of multiple genomic regions [1]. Drug response seems to be related to multifactorial and multigenic complex traits [15]. Therefore, the pharmacogenomics of antihypertensive drugs require future efforts to unravel additional genes and variants and to determine the epigenetic and regulatory pathways involved in the responsiveness to antihypertensive drugs [12]. The screening of individuals with undesired side effects could result in the optimization of treatment regimens and, therefore, in the improvement of patients’ outcomes and adherence to treatment. A pharmacogenomic strategy based on the choice of the most effective and well-tolerated drug regimen would be exceptionally valuable by requiring fewer drugs per patient, leading to greater cost-effectiveness and better blood pressure control, which would prevent cardiovascular and renal events and also improve the quality of life and longevity of hypertensive patients [1]. According to estimations, the approach based on genetically guided therapy for hypertension with the use of a multi-gene panel reduces total 3-year costs by 47%, and 89% of these savings are related to averting specific adverse events [19]. The results of studies and clinical practice confirm that antihypertension therapies based on the genotype are more effective, as they help to avoid major adverse events and decrease the costs of the treatment [19].

## Figures and Tables

**Table 1:** A summary of the results of the most interesting studies on CCBs presented in this review.

Drug	Population	Gene	Polymorphism	Result	Ref.
Verapamil	White, Hispanic, and Black hypertensive patients with cardiovascular disease (CAD)	*KCNMB1*	Glu65Lys (rs11739136)	Lys65 variant carriers achieved BP targets faster in comparison to Glu65Glu individuals (1.47 [interquartile range (IQR 2.77)] months in Lys65 carriers vs. 2.83 [(IQR) 4.17] months in Glu65Glu patients; *p* = 0.01).	[19]
			Val110Leu (rs2301149)	Leu110 allele decreased the risk of nonfatal myocardial infarction in patients treated with verapamil (HR 0.587, 95% confidence interval 0.33–1.04).	[19]
Verapamil	White, Hispanic, and Black hypertensive patients	*CACNA1C*	rs1051375A/A	46% reduction in the primary outcome in AA carriers treated with verapamil (OR 0.54, 95% CI 0.32–0.92), while the carriers of the GG genotype had higher risk of the composite primary outcome (OR 4.59 95% CI 1.67–12.67).	[25]
Verapamil	White, Black, and Hispanic	*CACNB2*	rs2357928	Carriers of the GG genotype had an increased risk of the primary outcomes if they were treated with verapamil compared to those on atenolol,	[25]
L-type dCCBs	Japanese Patients with essential hypertension	*CACNA1C* *CACNA1D*	rs527974G/A rs312481G/A rs3774426C/T	The combined presence of SNPs was associated with a considerable decrease in BP. GA + AA in CACNA1D rs312481G/A or with GG in CACNA1C 527974G/A—treatment non-responders.	[21]
Verapamil	White, Black, and Hispanic	*CYP3A5*	CYP3A5*6, rs10264272	CYP3A5 functional allele status was marginally associated with the SBP response to verapamil in Black (*p* = 0.075) and Hispanic (*p* = 0.056) but not in White (*p* = 0.40) populations; carriers of the two functional alleles had higher SBP. No association found with DBP response and CYP3A5 allele status.	[29]
Amlodipine	African American men and women with early hypertensive renal disease	*CYP3A4* *CYP3A4*	CYP3A4*1B, −392A/G, rs2740574	Female carriers of an A allele were over 3 times more likely to reach a target mean arterial pressure (MAP) of 107 mm Hg (the adjusted hazard ratio was 3.41 (1.20–9.64; *p* = 0.020)).	[32]
			T16090C (rs2246709)	Participants randomized to a lower MAP goal carrying the C allele were more likely to reach the target MAP (the adjusted hazard ratio was 2.04 (1.17–3.56; *p* = 0.010)).	[32]
Amlodipine	Healthy Korean males	*CYP3A5*	CYP3A5*3 (A6986G, rs776746)	Significant difference (20%) in the oral clearance of amlodipine between CYP3A5*1 carriers (27.0 ± 8.2 L/h) and CYP3A5*3/*3 carriers (32.4 ± 10.2 L/h) (*p* = 0.063). CYP3A5*1 carriers (3.8 ± 1.1 ng/mL) had considerably higher peak plasma concentrations compared to CYP3A5*3/*3 carriers (3.1 ± 0.8 ng/mL) (*p* = 0.037). No significant differences in BP and pulse rate were found between the studied groups.	[34]
Amlodipine	Chinese patients with mild to moderate essential hypertension	*ABCB1*	C3435T (rs1045642)	Amlodipine oral clearance (CL/F) was 1.5-fold higher in carriers of the TT genotype compared to other groups of carriers.	[35]

BP—blood pressure; IQR interquartile range; HR—hazard ratio; OR—odds ratio; Cl—confidence interval; SBP—systolic blood pressure; DBP—diastolic blood pressure.

**Table 2:** A summary of the results of the most interesting studies on angiotensin-II receptor blockers and angiotensin-converting enzyme inhibitors presented in this review.

Drug	Population	Gene	Polymorphism	Result	Ref.
Losartan	Population of GENRES, GERA II, and SOPHIA studies	*NPHS1*	349G/A (rs3814995)	SNP was associated with systolic (*p* = 2.0×10^−5^) and diastolic (*p* = 5.1×10^−4^) BP responses to losartan in GENRES, with systolic (*p* = 0.03) and diastolic (*p* = 0.02) BP responses in GERA II and diastolic BP responses (*p* = 0.03) in SOPHIA.	[7]
Enalapril	Patients with essential hypertension	*NOS3*	−665C/T (rs3918226)	Carries of the T allele for the functional tagSNP had more intense decreases in their blood pressure in response to enalapril at 20 mg/day.	[41]
Enalapril	Patients with essential hypertension	*NOS3*	tagSNP (rs3918188)	Patients carrying the AA genotype for tagSNP had lower decreases in blood pressure in response to enalapril. The TCA haplotype was associated with improved decreases in blood pressure in response to enalapril compared to the CAG haplotype.	[41]
Losartan	Subjects with mild-to-moderate essential hypertension	*CAMK1D*	rs10752271	Hypertensive carriers of the GG genotype for polymorphisms had a better BP response to losartan. Effect size of −5.5 ± 0.94 and a *p*-value of 1.2 × 10^-8^.	[42]
Candesartan	White and African Americans with primary hypertension	*SCNN1G*	rs11649420	GG genotype carriers had 3-fold greater BP response to candesartan compared to the combined group of AA+AG. The mean adjusted systolic BP/diastolic BP responses to candesartan were 7.0/5.5 mmHg greater for the GG group than for the AA+AG group.	[43]
Candesartan	White and African Americans with primary hypertension	*GPR83*	rs3758785	For the GG genotype, the odds of a good BP response to candesartan were more than 16-fold greater than those for the AA genotype. The mean adjusted systolic BP/diastolic BP responses to candesartan were 13.7/10.5 mmHg greater for the GG than for the AA genotype.	[43]
Enalapril	Brazilian hypertensive patients	*PRKCA*	rs16960228	The GA or AA genotypes and the A allele were associated with a lower reduction in mean BP and DBP after treatment and therefore with worse responses to enalapril compared to the GG genotype (*p* < 0.05).	[50]
Enalapril	Hypertensive patients	*NOS3*	−786T/C (rs2070744)	The TC/CC genotypes and the C allele for endothelial nitric oxide synthase were more frequent in good responders to enalapril treatment than in those who responded worse.	[52]
Enalapril	Hypertensive patients	*BDKRB2*	−58C/T (rs1799722)	The TT genotype occurred more frequently in individuals who responded better to enalapril compared to poor responders.	[52]

GERA—Genetic Epidemiology Research on Aging.

**Table 3:** A summary of the results of most interesting studies on the β-adrenergic antagonists presented in this review.

Drug	Population/Study Design	Gene	Polymorphism	Result	Ref.
Atenolol	White, Hispanic, and Black patients with hypertension and documented CAD	*ADRB1*	Ser49-Arg389	The presence of this haplotype was associated with a considerable risk of all-cause death among patients randomly assigned to verapamil SR (HR 8.58, 95% CI 2.06–35.8) but not atenolol (HR 2.31, 95% CI 0.82–6.55), suggesting a protective role for the β-blocker.	[59]
Atenolol	Participants of the SPS3 (Secondary Prevention of Small Subcortical Strokes) trial with hypertension	*ADRB1*	Ser49Gly (rs1801252)	Gly49 carriers treated with β-blockers had increased risk of adverse outcomes. Gly49 carriers treated with β-blockers had a 3-fold increased risk, while Gly49 carriers without β-blocker treatment had a 2-fold risk; thus, it seems that β-blocker treatment could amplify the effects on the Gly49 allele.	[60]
Metoprolol	White, African American, and Hispanic patients with hypertension	*ADRB1*	Arg389Gly (rs1801253)	Daytime diastolic BP responses to metoprolol among carriers of the Arg/Arg genotype were 3-fold greater compared to the Gly allele carriers (-13.3% +/- 8.4% versus -4.5% +/- 8.2%, *p* =.0018).	[61]
Carvedilol	Subjects with uncomplicated essential hypertension from the Jilin province of China	*ADRB1*	Arg389Gly (rs1801253)	In Chinese hypertensive 389Arg/Arg patients, treatment with carvedilol reduced BP to a greater extent (4-fold) than in individuals carrying the Gly allele (10.61 vs. 2.62 mm Hg, *p* = 0.013).	[62]
Metoprolol	Healthy individuals and patients with essential hypertension	*ADRB1*	Arg389Gly (rs1801253)	Subjects carrying the Gly/Gly genotype showed greater antihypertensive responses to metoprolol than the heterozygotes (*p* = 0.027).	[63]
Atenolol	Hypertensive Caucasians and African American participants from the PEAR trial	*GRK4*	A142V (rs1024323) R65L (rs2960306)	GRK4 65L and 142V variants, as well as the presence of the 65L-142V haplotype, significantly reduced the response to β-blocker monotherapy and also enhanced the risk of adverse long-term CV outcomes.	[66]
Atenolol	European American participants of the PEAR and PEAR-2 study	*FGD5*	rs294610	Carriers of the A allele had a considerably better BP response than non-carriers	[68]
Metoprolol Atenolol	African American hypertensive participants	*SLC25A31*	rs201279313	Heterozygotes versus the wild-type genotype had better diastolic BP responses to atenolol monotherapy, metoprolol monotherapy, and atenolol add-on therapy: −9.3 versus −4.6, −9.6 versus −4.8, and −9.7 versus −6.4 mm Hg, respectively (3-group meta-analysis *p* = 2.5×10(-8), β = −4.42 mm Hg per variant allele)	[73]
Atenolol	Finnish patients of the LIFE study	*ACY3*	rs2514036	Variation at the transcription start site of ACY3 was associated with a blood pressure response to atenolol in men	[74]

PEAR—Pharmacogenomic Evaluation of Antihypertensive Responses; CV—cardiovascular; LIFE—Losartan Intervention For Endpoint reduction in hypertension study.

**Table 4:** A summary of the results of the most interesting studies on diuretics presented in this review.

Drug	Population/Study Design	Gene	Polymorphism	Result	Ref.
Hydrochlorothiazide	South Korean patients	*ACE*	I/D rs1799752	A significant association was observed between the II and DD genotype and blood pressure changes (standard differences in means = 0.256; 95% CI, 0.109–0.403).	[94]
Hydrochlorothiazide	Never-treated individuals (Italy) with mild essential hypertension	*ACE* *ADD1*	I/D rs1799752 Gly460Trp (rs4961)	Carriers of the I/I genotype showed better antihypertensive responses to hydrochlorothiazide compared to those carrying the D/D genotype. A significantly greater blood pressure decrease was observed in carriers of the genotype ID or II+Gly460Trp or Trp460Trp compared to carriers of the genotype DD+Gly460Gly (−12.7 ± 1.9 mm Hg versus −3.43 ± 1.7 mm Hg) after chronic diuretic treatment.	[92]
Hydrochlorothiazide	South Korean patients	*ADD1*	Gly460Trp (rs4961)	A considerable relationship was revealed among the genotypes of Gly/Gly vs. Gly/Trp (standard differences in means= 2.78; 95% CI, 0.563–4.99) and Gly/Gly vs. Trp/Trp (standard differences in means = 1.80; 95% CI, 1.38–2.22).	[89]
Hydrochlorothiazide	Black and non-Hispanic white patients with essential hypertension	*GNB3*	C825T (rs5443)	The mean decreases in systolic and diastolic blood pressure were 6+/-2 (*p* < 0.001) and 5+/-1 (*p* < 0.001) mm Hg greater, respectively, in TT than in CC homozygotes.	[98]
Thiazide diuretic/β-blocker combination	White participants of the PEAR trial	*ALDH1A2*	rs261316	ALDH1A2 associated with an increased odds of having uncontrolled BP in combination therapy (odds ratio: 2.56, 95% confidence interval, 1.69–3.88, *p* = 8.64×10^-6^).	[101]
Hydrochlorothiazide	Non-Hispanic black subjects and non-Hispanic white subjects with essential hypertension	*LYZ, YEATS4, FRS2*	Combination of alleles for SNPs rs317689 (A), rs315135 (T), and rs7297610(C))	Carrying the ATC haplotype was associated with a much better diastolic BP response than persons with the ACT or ATT haplotype (nominal *p* = 2.39 × 10^-7^; Bonferroni corrected *p* = 0.024; simulated experiment-wise *p* = 0.040).	[103]
β-blocker or diuretic monotherapy	Hypertensive patients (DBP ≥ 100 mmHg) from the Nordic Diltiazem Study (NORDIL)	*NEDD4L*	rs4149601G/A	Carriers of the G-allele presented greater SBP reduction (19.5 ± 16.8 vs. 15.0 ± 19.3 mmHg, *p* < 0.001) and DBP reduction (15.4 ± 8.3vs. 14.1 ± 8.4 mmHg, *p* = 0.02). Carriers of the G-allele had greater protection from cardiovascular events [relative risk (RR) = 0.52, 95% confidence interval (CI) = 0.36–0.74, *p* < 0.001] compared to the AA homozygotes.	[110]
Hydrochlorothiazide	Patients from the PEAR clinical trial	*NEDD4L*	rs4149601 and rs292449	A significant relationship was found between increasing copies of the GC rs4149601-rs292449 haplotype and greater blood pressure responses to hydrochlorothiazide in white patients (*p* = 0.0006 and 0.006, SBP and DBP, respectively).	[114]
